# Contents of Hopes and Duties: A Linguistic Analysis

**DOI:** 10.3389/fpsyg.2018.00757

**Published:** 2018-05-16

**Authors:** Leigh Ann Vaughn

**Affiliations:** Department of Psychology, Ithaca College, Ithaca, NY, United States

**Keywords:** promotion, prevention, regulatory focus, goals, LIWC

## Abstract

People in a prevention focus tend to view their goals as duties and obligations, whereas people in a promotion focus tend to view their goals as hopes and aspirations. The current research suggests that people's attention goes to somewhat different experiences when they describe their hopes vs. duties. Two studies randomly assigned participants (*N* = 953) to describe a hope vs. duty. Specifically, Study 1 asked participants to describe a personal experience of pursuing a hope vs. duty, and Study 2 asked participants to describe a current hope vs. duty they had. I analyzed these descriptions with Linguistic Inquiry and Word Count (LIWC) 2015. Consistent with earlier research on regulatory focus, participants wrote more about positive outcomes when describing hopes and social relationships when describing duties. The current research suggests that the effectiveness of common regulatory focus and regulatory fit manipulations could depend on participants' freedom to choose the experiences they bring to mind when they describe their hopes and duties.

## Introduction

The following scenario is from a research participant who described pursuing a hope or aspiration – something he ideally wanted to do. In many ways, it is representative of what participants brought to mind about their hopes. “Earlier in the year, I was hiking on a long trail. This was in order to get to a campsite many miles away from the road. The hike was beautiful and once to the campsite I was in awe of the glory of nature surrounding me, as I had desired to see.”

The next scenario is from a research participant who described pursuing a duty or obligation—something she believed she ought to do. In many ways, it is representative of what participants brought to mind about their duties. “I had to drive about 30 min away simply to change my mother's tire. Seeing as how my mother is single, she depends on me and my husband a lot. It was after midnight when our phone rang saying that she cut a corner too sharp and hit the curb and her tire went flat.”

These examples suggest that people may refer to somewhat different kinds of experiences when they describe their hopes than when they describe their duties. Hopes and duties are goals that relate to different self-regulatory orientations, or regulatory foci. When people are in a promotion focus, they strive for growth and tend to view their goals as hopes and aspirations (Higgins, 1997, 1998). In contrast, when people are in a prevention focus, they strive for security and tend to view their goals as duties and obligations (Higgins, 1997, 1998). Much of the research on regulatory focus experimentally varies these self-regulatory orientations by asking participants to write about their hopes vs. duties (e.g., Freitas and Higgins, 2002; Cesario et al., 2004; Vaughn et al., 2006b). To date, no published research has examined whether there are systematic differences in the contents of these goals. Such research is important, because it would reveal whether people tend to bring to mind different kinds of activities and experiences when describing hopes vs. duties. One way to explore these possible differences is to examine the language people use to describe these goals, because language can reveal what people are paying attention to (e.g., Tausczik and Pennebaker, 2010; Pennebaker, 2011). If there were linguistic differences between descriptions of hopes and duties, then it would suggest that the effectiveness of common manipulations of regulatory focus that ask participants to write about their hopes or duties could depend on how much freedom participants have to choose the activities they bring to mind. As described next, existing research on regulatory focus suggests that descriptions of hopes and duties could differ in how much they refer to positive outcomes and social relationships.

### Regulatory focus and attention to positive outcomes

When people are in a promotion focus, they want to approach the presence of positive outcomes (gains) and avoid the absence of positive outcomes (nongains), and when people are in a prevention focus, they want to approach the absence of negative outcomes (nonlosses), and avoid the presence of negative outcomes (losses; Higgins, 1997, 1998). Approaching goals with eagerness helps sustain a promotion focus, whereas approaching goals with vigilance helps sustain a prevention focus (Higgins, 2000, 2005). Accordingly, selectively bringing positive information about the self to mind can sustain optimism and eagerness and is more common among people in a promotion focus, whereas selectively bringing negative information about the self to mind can sustain defensive pessimism and vigilance and is more common among people in a prevention focus (Scholer et al., 2014; also see Vaughn, 2017b). When people succeed at a gain, they experience more positive emotions than when people succeed at a nonloss (Idson et al., 2000). Research also shows that people recall more positive emotion in past goal pursuits that were promotion-focused rather than prevention-focused (Pattershall et al., 2012).

In short, research on regulatory focus shows that people who are in a promotion focus pay more attention to positive outcomes than people who are in a prevention focus and that people who are in a promotion focus experience these positive outcomes more intensely. This earlier research on regulatory focus suggests that, compared to descriptions of duties, descriptions of hopes could have more references to positive emotions and positive events.

### Regulatory focus and attention to managing social relationships

Research suggests that people often try to secure relationships with others by fulfilling their duties and obligations, and that they often try to grow and accomplish new things in life through being positively distinct from others (e.g., Lee et al., 2000). Research also suggests that people in an interdependent state of mind consider prevention-focused information more important than promotion-focused information, whereas people in an independent state of mind consider promotion-focused information more important than prevention-focused information (Lee et al., 2000; Aaker and Lee, 2001). Because managing social relationships often involves fulfilling duties and obligations, descriptions of duties could have more references to social relationships than descriptions of hopes.

### The current research

In light of these differences in what people pay attention to within experiences of promotion and prevention focus, there was reason to expect that there could be differences in the language people use to describe their hopes vs. duties. Language tracks focus of attention (Tausczik and Pennebaker, 2010; Pennebaker, 2011), and this link between language and attentional focus occurs in many settings. For example, research shows that if someone currently is being more honest, is lower in social status, or is more depressed, they are more likely to use first-person pronouns like *I, me* or *my*, because people often avoid self-focus when lying, focus on subordinates when high in status, and focus on themselves when depressed (e.g., Tausczik and Pennebaker, 2010; Pennebaker, 2011). Linguistic Inquiry and Word Count (LIWC; Pennebaker et al., 2015a) is software for analyzing linguistic features of writing or speech samples that assesses the percentages of words in writing samples that fit into various linguistic categories (e.g., first-person pronouns, emotion words, words about social processes, words about work). The current research is the first to use LIWC to study the language people use when describing their hopes and duties.

Two studies examined different kinds of writing about hopes and duties. Study 1 used text samples from participants who were randomly assigned to describe a personal experience of pursuing a hope or a duty. The data set for this study (Vaughn, 2017a) is from published research that tested hypotheses about relationships between regulatory focus theory (Higgins, 1997, 1998) and self-determination theory (Deci and Ryan, 2000). These relationships with self-determination theory are described elsewhere (Vaughn, 2017b) and are not the focus of the current research. Study 2 used text samples from participants who were randomly assigned to describe a current hope or duty. In both of these studies, I used LIWC 2015 (Pennebaker et al., 2015a) to provide word counts, and I tested for differences between promotion and prevention conditions. Additionally, I compared word usage in promotion and prevention conditions with base rates of word usage in other contexts reported by Pennebaker et al. (2015b): blogs, novels, natural speech, the *New York Times*, twitter, and expressive writing about traumatic or stressful events.

## Study 1

### Method

#### Dataset

The current study used the combined samples of Studies 1a-1c in Vaughn's (2017a,b) research, because these studies were the ones in which I randomly assigned participants to write about either a promotion-focused or prevention-focused personal experience. These samples' procedures were identical through the writing task. The data and materials for the earlier research are available for others to investigate (https://osf.io/uxneu). Additionally, the data files containing LIWC output and the materials for the current study are publicly available (https://osf.io/p8s6c/). The data files include .sav, .dat, and codebook files, as well as .docx files containing the writing samples.

I used Faul et al. (2007) software for power analyses. Additionally, I report all measures, manipulations, and exclusions in these studies through the page containing the writing samples that I analyzed in the current investigation.

#### Participants

Vaughn (2017b) recruited participants through Amazon's Mechanical Turk (Mturk) website. Eligible Mturk workers resided in the U.S. or Canada, had an approval rate of at least 95% on Mturk tasks, and had 500–5,000 approved tasks. Participants received $0.30 or $0.40, depending on the length of the study (~$0.10 per minute).

To discourage multiple responding, Vaughn (2017b) used Peer et al.'s (2012) procedure, the “Prevent Ballot Box Stuffing” option in Qualtrics, and TurkPrime. I only used the first response of any participant who responded more than once. Of the 617 responses collected in Vaughn's (2017a,b) Studies 1a-1c, I excluded three cases because of multiple responding. Additionally, I excluded the data from one participant who reported being <18 years old (she reported that her age was 2). I also excluded the data from five participants who did not do the writing task (three in duties, two in hopes). Moreover, I excluded the data from seven participants for whom the latitude/longitude data automatically collected by the survey indicated a location outside the U.S. or Canada.

After excluding 16 participants for the aforementioned reasons, the sample in Vaughn's (2017a,b) Studies 1a-1c and the current investigation contained 601 participants. This sample had slightly more women (51.08%, *n* = 307) than men (48.09%, *n* = 289; five participants reported “other” for gender or left this question blank). Mean age was 33.89 (*SD* = 11.30; range = 18–71). Participants were asked to select all the racial/ethnic categories to which they belonged; 79.04% selected White (*n* = 475), 7.82% selected African American (*n* = 47), 7.82% selected Asian (*n* = 47), 6.66% selected Hispanic, or Latina/Latino (*n* = 40), 1.83% selected multiethnic (*n* = 11), 1.33% selected Native American or Alaska Native (*n* = 8), and 0.67% selected “other” (*n* = 4). Most of the participants said they lived in the U.S. (99.30%, *n* = 597).

A power analysis showed that this combined sample of 601 participants (302 in the duties condition, 299 in the hopes condition) provides slightly more than 95% power to detect a between-condition difference of *d* = 0.30 at *p* = 0.05, two-tailed. For completeness, I will describe how I arrived at the sample sizes in the initial research (also see Vaughn, 2017a,b). In Study 1a, there were 105 participants (52 in hopes, 53 in duties). I chose the target sample size ahead of time based on a guess and the guideline of 50 participants per condition (Simmons et al., 2013). In Study 1b, there were 298 participants (146 in hopes, 152 in duties). I chose the target sample size ahead of time by conducting a power analysis using data from Study 1a and aiming for 95% power to detect the smallest expected difference between conditions, on the dependent variables of interest in the initial research (Vaughn, 2017a,b). In Study 1c, there were 198 participants (101 in hopes, 97 in duties). I chose the target sample size ahead of time by conducting a power analysis using data from the combined sample of Studies 1a and 1b and aiming for 80% power to detect the smallest expected difference between conditions on the dependent variables of interest in the initial research.

#### Materials and procedure

The studies reported as Study 1 were carried out in accordance with the recommendations of the Ithaca College Institutional Review Board with written informed consent from all subjects. All participants gave written informed consent in accordance with the Declaration of Helsinki. The Institutional Review Board of Ithaca College approved the protocols of these studies.

The first page of stimulus materials randomly assigned participants to write about either a promotion-focused experience (“You were doing what you ideally wanted to, in order to fulfill a hope or aspiration you had”) or a prevention-focused experience (“You were doing what you believed you ought to, in order to fulfill a duty or obligation you had”). Participants' text samples were from this page. The procedures of Vaughn's (2017a,b) Studies 1a-1c were identical through the page with the writing task. Because the studies did not have a back button, participants' subsequent responses could not have affected what they wrote on the first page. After several other pages of materials (described by Vaughn, 2017a,b), participants arrived at a page where they provided demographic information including age, gender, ethnicity, and state of residence. Finally, participants received a debriefing page and a code to use for indicating they had done the study on MTurk.

#### Linguistic analyses

I analyzed the writing samples using the LIWC 2015 program (Pennebaker et al., 2015a). LIWC calculates the percentages of words in a writing sample that match up with particular categories. For most of the categories, mean values indicate the mean percentages of all of the words that participants used that fell into a particular category. For example, a mean score of 5.43 for positive emotion words indicates that 5.43% of the words participants used were associated with positive emotion (e.g., *love, nice, sweet*). The exceptions were the mean values for word count, words per sentence, and the summary variables (analytical/categorical vs. dynamic thinking, authenticity, clout/status, and emotional tone). To arrive at these summary variables for LIWC 2015, Pennebaker et al. (2015a) derived summary indexes from their lab's previous research and converted them to percentiles based on standardized scores from large samples of writing from comparison groups. The analytical/categorical vs. dynamic thinking variable is by Pennebaker et al. (2014), the authenticity variable is by Newman et al. (2003), the clout/status variable is by Kacewicz et al. (2013), and the emotional tone variable is by Cohn et al. (2004). In the results section, I will describe in detail the summary variables that showed significant differences. I prepared the writing samples for LIWC by running each writing sample through Word's standard spell-check program and correcting spelling errors, which were rare.

### Results

There are 92 word categories in the LIWC 2015 standard dictionary. (For descriptions of all categories, see Pennebaker et al., 2015b). In the current research, I used 73 of these categories, omitting the word categories used at extremely low rates – <0.5% of the time^1^.

In the following analyses, positive *t*-values indicate higher scores in the hopes condition. The average word count was 35.74 (*SD* = 28.73), and it did not differ significantly between conditions, *t*(599) = −1.45, *p* = 0.149. To limit the potential for false-positive results, I set a conservative limit for reporting of all other results. The Bonferoni correction to *p* < 0.05 for 73 tests is *p* < 0.00069, so I set the cut-off for significance at *p* < 0.001, two-tailed, with the additional requirement of *d* > 0.35. (In this sample, *p* = 0.001 corresponded to *d* = ± 0.28).

Table 1 presents condition descriptive statistics and tests of between-condition differences for the LIWC categories on which there were significant differences between hopes and duties^2^ Table 2 presents means for the hopes and duties conditions along with base rates of word usage in other forms of writing (Pennebaker et al., 2015b)^3^.

**Table 1:** Study 1: condition statistics and tests of significant between-condition differences.

		**Hopes**		**Duties**		**Tests of between–condition differences**				
**Word type**	**Examples**	***M***	***SD***	***M***	***SD***	***df***	***t***	**Mean diff**.	**95% CI**	***d***
**HOPES HIGHER**										
Analytical/categorical vs. dynamic	^a^	69.01	28.30	53.94	32.77	588.19	6.04	15.07	[10.17, 19.98]	0.49
Emotional tone	^b^	61.77	34.27	43.13	34.48	599.00	6.64	18.63	[13.13, 24.14]	0.54
Positive emotion	Love, nice, sweet	3.82	4.32	2.27	3.06	536.24	5.09	1.56	[0.96, 2.16]	0.42
Achievement	Win, success, better	4.38	5.38	2.38	3.85	539.66	5.24	2.00	[1.25, 2.75]	0.43
Reward	Take, prize, benefit	3.56	5.06	1.80	2.83	466.50	5.24	1.75	[1.10, 2.41]	0.43
Work	Job, majors, xerox	9.72	9.27	5.88	6.55	535.95	5.87	3.84	[2.56, 5.13]	0.48
Leisure	Cook, chat, movie	2.89	5.64	0.90	2.09	377.53	5.73	1.99	[1.31, 2.67]	0.47
**DUTIES HIGHER**										
Clout/status	^c^	24.73	23.57	34.59	30.00	569.65	−4.48	−9.86	[−14.19, −5.54]	−0.37
Words per sentence	–	15.52	6.68	18.26	8.05	599.00	−4.53	−2.73	[−3.92, −1.55]	−0.37
Total function words	It, to, no, very	53.33	10.33	58.40	8.46	599.00	−6.58	−5.07	[−6.58, −3.55]	−0.54
Total pronouns	I, them, itself	15.54	7.13	19.24	6.93	599.00	−6.45	−3.70	[−4.83, −2.57]	−0.53
Personal pronouns	I, them, her	12.31	6.02	14.80	6.02	599.00	−5.07	−2.49	[−3.46, −1.53]	−0.41
Third person singular	She, her, him	0.20	1.07	1.86	3.48	358.05	−7.93	−1.66	[−2.08, -1.25]	−0.65
Conjunctions	And, but, whereas	4.69	4.05	6.62	4.47	599.00	−5.55	−1.93	[−2.62, -1.25]	−0.45
Negations	No, not, never	0.42	1.10	1.27	2.13	452.50	−6.16	−0.85	[−1.12, −0.57]	−0.50
Negative emotion	Hurt, ugly, nasty	0.41	1.37	1.53	2.95	426.97	−5.98	−1.12	[−1.49, −0.75]	−0.49
Social processes	Mate, talk, they	4.39	6.23	9.82	8.70	545.33	−8.82	−5.44	[−6.65, −4.22]	−0.72
Family	Daughter, dad, aunt	0.70	2.64	2.01	3.65	548.25	−5.04	−1.31	[−1.82, −0.80]	−0.41
Friends	Buddy, neighbor	0.15	0.93	0.92	2.70	371.71	−4.63	−0.76	[−1.09, −0.48]	−0.38
Female references	Girl, her, mom	0.31	1.60	2.03	4.30	383.37	−6.49	−1.72	[−2.24, −1.19]	−0.53
Male references	Boy, his, dad	0.41	1.70	1.38	3.54	433.92	−4.29	−0.97	[−1.41, −0.52]	−0.35
Differentiation	Hasn't, but, else	1.31	2.35	2.67	3.48	528.34	−5.66	−1.37	[−1.84, −0.89]	−0.46
Affiliation	Ally, friend, social	1.45	3.06	3.39	5.24	486.33	−5.55	−1.94	[−2.63, −1.25]	−0.45

*To limit the potential for false-positive results, I set a conservative limit for inclusion in this table at p < 0.001, two-tailed, and d ≥ 0.35. Degrees of freedom are adjusted for heterogeneity of variance. Mean values indicate the mean percentage of all of the words that participants used that fell into a particular category, except the mean values for words per sentence and the summary variables (analytical thinking, tone, and clout)*.

a *The analytical/categorical thinking variable is by Pennebaker et al. (2014)*.

b *The emotional tone variable is by Cohn et al. (2004)*.

c *The clout/status variable is by Kacewicz et al. (2013)*.

**Table 2:** Study 1: Comparison of writing in the current research with other forms of linguistic expression (Pennebaker et al., 2015b).

	**Means from current research**		**Means from Pennebaker et al. (****2015b****)**					
**Word type**	**Hopes**	**Duties**	**Blogs**	**Expressive writing**	**Novels**	**Natural speech**	**NY times**	**Twitter**
**HOPES HIGHER**								
Analytical/categorical vs. dynamic	69.01	53.94	49.89	44.88	70.33	18.43	92.57	61.94
Emotional tone	61.77	43.13	54.50	38.60	37.06	79.29	43.61	72.24
Positive emotion	3.82	2.27	3.66	2.57	2.67	5.31	2.32	5.48
Achievement	4.38	2.38	1.27	1.37	0.91	0.99	1.82	1.45
Reward	3.56	1.80	1.49	1.56	1.04	1.73	1.07	1.86
Work	9.72	5.88	2.04	2.64	1.20	2.87	4.49	2.16
Leisure	2.89	0.90	1.50	1.17	0.56	1.11	1.67	2.11
**DUTIES HIGHER**								
Clout/status	24.73	34.59	47.87	37.02	75.37	56.27	68.17	63.02
Words per sentence	15.52	18.26	18.40	18.42	16.13	–	21.94	12.10
Total function words	53.33	58.40	53.10	58.27	54.51	56.86	42.39	46.08
Total pronouns	15.54	19.24	16.20	18.03	15.15	20.92	7.41	13.62
Personal pronouns	12.31	14.80	10.66	12.74	10.35	13.37	3.56	9.02
Third person singular	0.20	1.86	1.50	2.01	4.80	0.77	1.53	0.64
Conjunctions	4.69	6.62	6.43	7.46	6.28	6.21	4.85	4.19
Negations	0.42	1.27	1.81	1.69	1.68	2.42	0.62	1.74
Negative emotion	0.41	1.53	2.06	2.12	2.08	1.19	1.45	2.14
Social processes	4.39	9.82	8.95	8.69	12.26	10.42	7.62	10.47
Family	0.70	2.01	0.46	0.77	0.39	0.31	0.33	0.36
Friends	0.15	0.92	0.40	0.55	0.25	0.37	0.18	0.43
Female references	0.31	2.03	0.91	1.37	1.88	0.55	0.62	0.54
Male references	0.41	1.38	1.31	1.47	4.09	0.80	1.38	0.84
Differentiation	1.31	2.67	3.31	3.40	2.82	3.73	2.03	2.62
Affiliation	1.45	3.39	2.20	2.45	1.39	2.06	1.69	2.53

The following examples are representative of the linguistic differences between the hopes and duties conditions. The first four examples are from the hopes condition, where participants described a personal experience of pursuing a hope or aspiration—something they ideally wanted to do:

Once, I wanted to learn how to sew. I bought a sewing machine and started researching on the internet. I started out small and sewed a dress. Now, I am able to sew very well and make some extra money from learning to do this.
I went through 4 years of university to get a computer engineering degree. It was important for me to get a degree in something that I could see myself doing for a long time and also knew would be helpful in finding a job.
I wanted to make a really nice wedding gift so that my son and his wife would have a unique gift. I decided to buy a clear vase and make origami boxes and butterflies out of dollar bills to fill it up.
I took my bike to Belgium and rode around the country visiting breweries. I learned about beer, drank good beer, and got a lot of exercise. I was able to experience the culture of the country and learn about its history.

The second four examples are from the duties condition, where participants described a personal experience of pursuing a duty or obligation—something they believed they ought to do:

We went and spent Mother's Day over at my husband's grandmother's house. I would have rather stayed home but we knew that she needed the company and it would make her feel better to get to see our daughters and my husband.
I followed the orders my boss gave me at work, which were to call all of our clients and recommend a new premium membership we offer. This was my obligation, and I did what I believed I ought to within that day.
I stayed late at work to rewrite our schedule because two of our doctors were fired. I could have waited until later to do this but it seemed like I should take care of it early on my own time to avoid confusion.
I was babysitting my younger cousin who is 5 years old, and I refused to let her eat packaged ramen noodles for lunch because I thought it was unhealthy. She got upset, but I ignored it because I believe her physical health was more important.

These examples and the results below indicate that participants referred more to positive outcomes and emotions in descriptions of pursuing hopes, and they referred more to social relationships in descriptions of pursuing duties. Below, I summarize results of the statistical analyses of the words participants used to describe pursuing hopes and duties, and I compare these results to the base rates of word usage that Pennebaker et al. (2015b) reported. All of the correlations that I present below were significant at *p* < 0.001, including the weakest correlations.

#### Analytic/categorical vs. dynamic writing

Participants who described pursuing a hope wrote more about categories and things, whereas participants who described pursuing a duty wrote more about dynamic interpersonal processes. Analytical/categorical writing “methodically defines and categorizes thoughts” and reflects the kind of writing that is often rewarded in college (Pennebaker, 2011, p. 286), whereas dynamic writing “is far more personal and works to tell a story” that is about action and changes, often in social relationships (Pennebaker, 2011, p. 297; also see Pennebaker et al., 2014). Compared to writing about hopes, writing about duties was more dynamic—more narrative and more about interpersonal processes. In contrast, writing about hopes was more categorical—more focused on objects, things, and categories and less focused on stories about interactions with other people. Means on the analytical/categorical vs. dynamic variable were moderately high in the hopes and duties conditions compared to the base rates that Pennebaker et al. (2015b) reported.

In addition to the findings displayed in Tables 1, 2, it is useful to examine correlations with specific categories of words. The analytical/categorical vs. dynamic variable related most strongly to function words (*r* = −0.45) in ways that reflected writing that was largely impersonal (e.g., infrequent use of personal pronouns, *r* = −0.53) and that was highly structured (frequent use of articles like *a, an*, and *the, r* = 0.48; and prepositions like *to, with*, and *above, r* = 0.62).

#### Emotional tone, including positive emotion, and negative emotion

Descriptions of pursuing hopes were more positive in emotional tone than descriptions of pursuing duties. The emotional tone variable reflects the difference between the use of positive and negative emotion words (Cohn et al., 2004; Pennebaker et al., 2015b). In the current research, scores on the emotional tone variable correlated strongly with use of positive emotion words (*r* = 0.76) and less strongly with use of negative emotion words (*r* = −0.40). Emotional tone did not correlate with any non-emotion word types more strongly than *r* = ± 0.21. The emotional tone of both hopes and duties was moderate compared to the base rates that Pennebaker et al. (2015b) reported. For example, the emotional tone of duties was about the same as the base rate in the *New York Times*.

#### Words about achievement, reward, and work

Participants who described pursuing a hope referred more to achievement, reward, and work than participants who described pursuing a duty. Additionally, references to achievement, reward, and work were more frequent in descriptions of either hopes or duties than in any of the base rates that Pennebaker et al. (2015b) reported. Achievement related positively to reward (*r* = 0.33) and to work (*r* = 0.44), and reward related positively to work (*r* = 0.24). Outside of relationships with reward and work, achievement words correlated most strongly with use of words containing six letters or more (*r* = 0.26). Use of big words with six letters or more is part of the analytical/categorical summary variable and reflects more analytical/categorical thinking (Pennebaker, 2011; Pennebaker et al., 2014). In contrast, outside of relationships with achievement and work, words about reward did not correlate with use of any other word types more strongly than *r* = ± 0.21. Outside of relationships with achievement and reward, words about work correlated most strongly with use of words containing six letters or more (*r* = 0.38).

#### Words about leisure

References to leisure were more frequent in descriptions of hopes than in any of the base rates that Pennebaker et al. (2015b) reported, whereas they were moderately frequent in descriptions of duties. Leisure words did not correlate with use of any other word types more strongly than *r* = ± 0.21, except for function words (*r* = −0.215).

#### Social processes and affiliation

Participants who described pursuing a duty referred more to affiliation and social processes than participants who described pursuing a hope. Additionally, references to social processes were moderately frequent in descriptions of both hopes and duties compared to the base rates that Pennebaker et al. (2015b) reported. In contrast, affiliation words were more frequent in descriptions of duties than any of the base rates that Pennebaker et al. (2015b) reported, whereas affiliation words were less frequent in descriptions of hopes than in any of these base rates except for novels.

Words about social processes and affiliation were highly correlated (*r* = 0.62). Besides affiliation, social processes correlated most strongly with the clout/status variable (*r* = 0.76) and with third-person singular pronouns such as *she, her*, and *him* (*r* = 0.51).

Within the category of words about social processes, affiliation correlated most strongly with family (*r* = 0.36) and friends (*r* = 0.51), but it did not correlate strongly with words about females (*r* = 0.17) or males (*r* = 0.16). Outside of words about social processes, affiliation correlated most strongly with first-person plural pronouns like *we, us*, and *our* (*r* = 0.35) and words about home such as *kitchen* and *landlord* (*r* = 0.25).

#### Function words, including pronouns

Participants who described pursuing a duty used more function words than participants who described pursuing a hope. Function words include pronouns (e.g., *I, she, it*), articles (e.g., *a, an, the*), prepositions (e.g., *up, with, in, for*), auxiliary verbs (e.g., *is, don't, have*), negations (e.g., *no, not, never*), conjunctions (e.g., *but, and, because*), quantifiers (e.g., *few, most, some*), and common adverbs (e.g., *very, really*). In contrast to words like *grandmother, client, school*, and *bike*, which convey what someone is talking about, the precise meaning of statements like, “She met us there earlier,” are impossible to understand without knowing some things about the writer's social context (Tausczik and Pennebaker, 2010; Pennebaker, 2011). Thus, people use function words more often when they give some context for the actors' thoughts, feelings, and behaviors, which tends to happen in narratives about dynamic social interactions (e.g., Pennebaker, 2011).

Results on function words are consistent with those on social processes, affiliation, and the analytic/categorical vs. dynamic variable. That is, when describing experiences of pursuing duties, participants were more likely to use function words, which is consistent with telling stories about dynamic social interactions. Function words were moderately frequent in descriptions of duties and hopes compared to the base rates that Pennebaker et al. (2015b) reported.

Overall use of function words correlated most strongly with these specific types of function words: pronouns (*r* = 0.54), personal pronouns (*r* = 0.34), indefinite pronouns such as *its, it's*, and *those* (*r* = 0.46), conjunctions (*r* = 0.34), *p* < 0.001, and auxiliary verbs such as *am, will*, and *have* (*r* = 0.58). Besides relationships with other function words, overall use of function words related most strongly to the analytical/categorical vs. dynamic variable (*r* = −0.45), indicating more use of function words in more dynamic, less categorical language.

#### Clout/status

Participants who described pursuing a duty scored higher on the clout/status variable than participants who described pursuing a hope. However, this finding likely reflects differences in attention to social relationships more than differences in social status.

When people engage in face-to-face or written communication, those who are higher in status pay more attention to their audience than those who are lower in status, which is evident in the words that they use (Kacewicz et al., 2013). Specifically, people higher in the social hierarchy use fewer first-person pronouns (*I, me, my*), more first-person plural pronouns (*we, us, our*), and more second-person pronouns (*you, your*) than people lower in the social hierarchy.

In contrast to the writing samples in Kacewicz et al.'s (2013) research, the writing samples in the current research were not from people engaging in a conversation. Additionally, the hopes and duties means on the clout/status variable were very low, and they were closest to the base rate in expressive writing about a personal experience. Participants who wrote about pursuing either hopes or duties used first-person pronouns a lot, and they used first-person plural pronouns much less. They almost never used second-person pronouns. This pattern of pronoun use, if observed in a conversation between two people, would suggest that the speaker is low in status. However, in the current research, this pattern of pronoun use is more consistent with writing about a personal experience. Besides first-person pronouns (*r* = −0.53), the clout/status variable related most strongly to words about social processes (*r* = 0.76) and affiliation (*r* = 0.48).

#### “Exclusive” words: conjunctions, negations, and differentiation

Participants who described pursuing a duty used more exclusive words than participants who described pursuing a hope. People use more conjunctions, negations, and differentiation words when they are telling a story in which they distinguish between what happened and what did not, what they thought and what they did not, and what belongs in a category and what does not (e.g., Pennebaker, 2011). Compared to descriptions of pursuing hopes, descriptions of pursuing duties thus were more likely to be stories about dynamic social interactions in which participants distinguished between what they actually did and what they could have done, what they may have wanted to do, or what someone else wanted them to do. (For example, “I would have rather stayed home but we knew that she needed the company…” “I could have waited until later to do this but it seemed like I should take care of it early on my own time to avoid confusion.” “She got upset, but I ignored it because I believe her physical health was more important.”) Compared to the base rates that Pennebaker et al. (2015b) reported, conjunctions, negations, and differentiation words moderately frequent in descriptions of duties and very infrequent in descriptions of hopes.

Conjunctions related to negations (*r* = 0.15) and differentiation words (*r* = 0.24), and negations related to differentiation words (*r* = 0.61). The strongest correlations between these word types and other linguistic categories were with the analytic/categorical vs. dynamic variable (*r*s = −0.55, −0.32, and −0.35 for conjunctions, negations, and differentiation words, respectively) and with words about cognitive processes, which include *cause, know*, and *ought* (*r*s = 0.24, 0.41, and 0.61 for conjunctions, negations, and differentiation words, respectively). In short, exclusive words were more frequent in writing that was more dynamic and more about cognitive processes.

#### Words per sentence

Participants who described pursuing duties used longer, more complex sentences than participants who described pursuing hopes. Additionally, the number of words per sentence in descriptions of both duties and hopes was moderate compared to the base rates that Pennebaker et al. (2015b) reported. Words per sentence related most strongly to function words (*r* = 0.36) and among function words, words per sentence related most strongly to conjunctions (*r* = 0.26).

### Discussion

This is the first investigation of the activities and experiences people describe when they write about their hopes vs. duties. The study randomly assigned participants to write about personal experiences of pursuing a hope or aspiration vs. a duty or obligation, and LIWC 2015 (Pennebaker et al., 2015a) provided word counts. Linguistic analysis showed systematic differences between descriptions of hopes and duties that are consistent with what research on regulatory focus would suggest (e.g., Higgins, 1997, 1998; Lee et al., 2000; Aaker and Lee, 2001). Specifically, descriptions of hopes were more about positive outcomes (e.g., as reflected in positive emotional tone and references to reward and achievement), whereas descriptions of duties were more about maintaining social relationships (e.g., as reflected in references to social processes and affiliation).

A noteworthy similarity in what participants described is that they often wrote about achievement, reward and work. Frequencies of these words were higher in descriptions of both hopes and duties than base rates of these words in blogs, novels, natural speech, the *New York Times*, twitter, and expressive writing about traumatic or stressful events (Pennebaker et al., 2015b). Achievement, reward, and work are linguistic categories that people may commonly bring to mind when describing personal experiences of goal pursuit, regardless of the self-regulatory state in which they pursued the goal. If people tend to direct their attention to achievement, reward, and work in any self-regulatory state, then the use of these linguistic categories could be frequent in descriptions of other self-regulatory experiences.

A potential limitation on the generalizability of the results from Study 1 is the type of writing participants did: describing a personal experience of pursuing a hope vs. a duty. There are many ways to write about hopes and duties, and they may not produce the same kinds of differences as in Study 1. The main goal of Study 2 was to assess how well the results of Study 1 generalize to a different form of writing: describing a current, important hope vs. duty.

## Study 2

A commonly used method of varying regulatory focus is to randomly assign participants to describe their current hopes vs. duties (e.g., Freitas and Higgins, 2002; Vaughn et al., 2006a, 2008, 2013). Study 2 examined the generalizability of Study 1's results to this more constraining type of writing instruction. I expected that descriptions of hopes would be more about positive outcomes and descriptions of duties would be more about social relationships.

### Method

The data files containing LIWC output and the materials for Study 2 are publicly available (osf.io/p8s6c/). I used Faul et al.'s (2007) software for power analyses, and I report below all measures, manipulations, and exclusions in this study.

#### Participants

I recruited participants through the Mturk website, and they received $0.30 for this 3-min study. Participant qualifications and exclusion criteria for Study 2 were the same as in Study 1. I excluded data from one participant who did not do the writing task, from seven participants for whom the latitude/longitude data automatically collected by Qualtrics indicated that they were not in the U.S. or Canada (*n* = 12), and from ten cases in which the participants had already done the study. The sample had slightly more men (50.60%, *n* = 178) than women (49.40%, *n* = 174). Mean age was 33.22 (*SD* = 10.67; range = 18–73). Participants were asked to select all the racial/ethnic categories to which they belonged; 74.43% selected White (*n* = 262), 10.51% selected Asian (*n* = 37), 9.38% selected African American (*n* = 33), 7.10% selected Hispanic or Latino/Latina (*n* = 25), 2.56% selected multiethnic (*n* = 9), 1.14% selected Native American or Alaska Native (*n* = 4), 0.85% selected Native Hawaiian or Pacific Islander (*n* = 3), and 0.57% selected “other” (*n* = 2). Most of the participants said they lived in the U.S. (99.40%, *n* = 350). A power analysis indicated that this sample of 352 participants has 80% power to detect a between-condition difference of *d* = 0.30.

#### Materials and procedure

As with Study 1, Study 2 was carried out in accordance with the recommendations of the Ithaca College Institutional Review Board with written informed consent from all subjects. All participants gave written informed consent in accordance with the Declaration of Helsinki. The Institutional Review Board of Ithaca College approved the protocols of these studies.

The first page of stimulus materials was titled “An Important Goal You Have,” and it asked participants to “Please take a minute or so and write about a goal you have that is like the following description.” This page randomly assigned participants to write about either a duty (“This goal is a duty or obligation for you—it is something you believe you ought to do.”) or a hope (“This goal is a hope or aspiration for you—it is something you ideally would like to do.”). On the second page, participants answered a few questions about the goal they described^4^. The study did not have a back button, so participants' responses to questions on any subsequent pages could not have affected what they wrote on the first page. Participants then received a page where they provided demographic information including age, gender, ethnicity, and state of residence. Finally, they received a debriefing page and a code to use for indicating they had done the study on MTurk.

To prepare the writing samples for LIWC 2015, I ran each writing sample through Word's standard spell-check program and corrected spelling errors. As in Study 1, such errors were rare.

### Results

In the current research, I used 69 of the 92 categories in LIWC 2015, omitting the word categories used at extremely low rates – < 0.5% of the time^5^. Positive *t*-values in the following analyses indicate higher scores in the hopes condition. The average word count was 21.73 (*SD* = 17.70), and it did not differ significantly between conditions, *t*(350) = −0.06, *p* = 0.951. To limit the potential for false-positive results, I set a conservative limit for reporting of all other results. The Bonferoni correction to *p* < 0.05 for 69 tests is *p* < 0.00072, so I set the cut-off for significance at *p* < 0.001, two-tailed, with the additional requirement of *d* > 0.40. (In this sample, *p* = 0.001 corresponded to *d* = ± 0.34.)

Table 3 presents condition descriptive statistics and tests of between-condition differences for the LIWC categories on which there were significant differences between hopes and duties. In contrast to the 23 significant differences between descriptions of hopes and duties in Study 1, there were only four significant differences between hopes and duties in Study 2.

**Table 3:** Study 2: Condition statistics and tests of significant between-condition differences.

		**Hopes**		**Duties**		**Tests of between-condition differences**				
**Word type**	**Examples**	***M***	***SD***	***M***	***SD***	***df***	***t***	**Mean diff**.	**95% CI**	***d***
**HOPES HIGHER**										
Emotional tone	^a^	69.49	35.57	54.62	37.34	350.00	3.83	14.87	[7.23, 22.52]	0.41
Discrepancy	Should, would	4.77	4.47	2.61	3.83	340.89	4.86	2.16	[1.29, 3.03]	0.53
**DUTIES HIGHER**										
Social processes	Mate, talk, they	2.27	4.32	4.78	7.03	293.11	−4.04	−2.51	[−3.73, −1.28]	−0.43
Family	Daughter, dad, aunt	0.44	1.74	1.87	3.98	241.55	−4.37	−1.43	[−2.07, −0.78]	−0.47

*To limit the potential for false-positive results, I set a conservative limit for statistical significance, as in Study 1: p < 0.001, two-tailed, and d ≥ 0.40. Degrees of freedom are adjusted for heterogeneity of variance. Mean values indicate the mean percentage of all of the words that participants used that fell into a particular category, except the mean values for the summary variable (emotional tone)*.

a *The emotional tone variable is by Cohn et al. (2004)*.

Additionally, I examined which linguistic categories showed differences with Cohen's *d*s of 0.34 to 0.40 in Study 2, because the lower bound on Cohen's *d* in Study 1 was 0.34. There was only one such category: total pronouns (*d* = −0.37)^6^. Table 4 presents means for the hopes and duties conditions along with base rates of word usage in other forms of writing (Pennebaker et al., 2015b)^7^^,^ ^8^.

**Table 4:** Study 2: Comparison of writing in the current research with other forms of linguistic expression (Pennebaker et al., 2015b).

	**Means from current research**		**Means from Pennebaker et al. (****2015b****)**					
**Word type**	**Hopes**	**Duties**	**Blogs**	**Expressive writing**	**Novels**	**Natural speech**	**NY times**	**Twitter**
**HOPES HIGHER**								
Emotional tone	69.49	54.62	54.50	38.60	37.06	79.29	43.61	72.24
Discrepancy	4.77	2.61	1.56	1.74	1.48	1.45	0.89	1.54
**DUTIES HIGHER**								
Social processes	2.27	4.78	8.95	8.69	12.26	10.42	7.62	10.47
Family	0.44	1.87	0.46	0.77	0.39	0.31	0.33	0.36

The following examples are representative of the linguistic differences between the hopes and duties conditions. The first five examples are from the hopes condition, where participants described a current, important hope or aspiration—something they ideally wanted to do:

I would like to publish a non-fiction book in the next year.
I would like to pay off all of my debt and be debt-free.
I would ideally like to retire, after purchasing a hair salon and working from home.
I would like to make a career or earn money by doing art or music.
I would like to run a full marathon. I think it would be great to complete it.

The second five examples are from the duties condition, where participants described a current, important duty or obligation—something they believed they ought to do:

I have a goal to spend more time with my family this year.
Learn how to make money from home to have more time with my daughter.
My goal is to teach my children to swim by the end of next summer.
My goal is to get my budget in order and that works for my family.
I have to do what is right and follow my heart when it comes to relationships.

As in Study 1, these examples and the results below indicate that participants referred more to positive outcomes and emotions in descriptions of pursuing hopes, and they referred more to social relationships in descriptions of pursuing duties. Below, I summarize results of the statistical analyses of the words participants used to describe their hopes vs. duties, and I compare these results to the base rates of word usage that Pennebaker et al. (2015b) reported. All of the correlations reported below were significant at *p* < 0.001.

#### Emotional tone

Even though descriptions of hopes were more positive than descriptions of duties, the emotional tone in both conditions was moderately positive compared to base rates in other types of writing (Pennebaker et al., 2015b). Scores on the emotional tone variable correlated most strongly with positive emotion words (*r* = 0.70), affect words (*r* = 0.35), and negative emotion words (*r* = −0.31).

#### Discrepancy

Use of discrepancy words was more frequent in descriptions of either hopes or duties than in any of the base rates that Pennebaker et al. (2015b) reported, reflecting that participants wrote about something they would ideally like to do or believed they should do. However, participants in the hopes condition used discrepancy words more frequently. Discrepancy words related most strongly to first-person pronouns (*r* = 0.35) and to words about cognitive processes, which include *cause, know*, and *ought* (*r* = 0.47).

#### Social processes

Even though references to social processes were more frequent in descriptions of duties than descriptions of hopes, they were less frequent in descriptions of either hopes or duties than in any of the base rates that Pennebaker et al. (2015b) reported. Words about social processes related most strongly to words about affiliation (*r* = 0.55), the clout/status variable (*r* = 0.59), and words about family (*r* = 0.57).

#### Family

References to family were more frequent in descriptions of duties than in any of the base rates that Pennebaker et al. (2015b) reported, whereas references to family were less frequent in descriptions of hopes than in any of these base rates. Besides words about social processes, words about family correlated most strongly with words about affiliation (*r* = 0.61).

### Discussion

As expected, Study 2 showed that descriptions of current hopes were more about positive outcomes than descriptions of current duties were, and descriptions of current duties were more about social relationships than descriptions of current hopes were. Additionally, descriptions of hopes and duties differed on fewer dimensions than in Study 1, with significant differences on only four linguistic categories (vs. 23 categories in Study 1). In Study 2, emotional tone was more positive and discrepancy words were more common in description of hopes, whereas references to social processes and family were more common among participants who described duties.

While Study 2 corroborates the basic findings of Study 1, it also suggests that the language categories that differ between descriptions of hopes and duties depend on the kind of writing participants do. Study 2 constrained participants to write about a current hope or duty. Accordingly, use of words with a present focus, such as *today, is*, and *now*, were more frequent in Study 2 (*M* = 14.99) than in Study 1 (*M* = 7.29). Participants' stronger focus on the present may have contributed to more frequent references to money in Study 2 (*M* = 4.76) than in Study 1 (*M* = 1.49) or in any of the base rates that Pennebaker et al. (2015b) reported (base rate *M*s = 0.41 to 1.47). Participants in Studies 1 and 2 were MTurk workers who received monetary compensation for doing the study. However, Study 1 asked participants about a personal experience they have had. It appears that participants in Study 2 thought more about their current reasons for going to Mechanical Turk, which for most or all of MTurk workers include making money (Paolacci et al., 2010; Ross et al., 2010). Whether this is the only reason why descriptions of hopes and duties differed less in Study 2 than in Study 1 is not clear, because the writing samples in Study 2 were also shorter. With shorter writing samples, there would be less variation in the frequencies of word types for LIWC to detect.

As in Study 1, a notable similarity between descriptions of hopes and duties was the high frequency of references to achievement (*M* = 5.31), reward (*M* = 4.70), and work (*M* = 9.49) relative to the base rates of these words that Pennebaker et al. (2015b) reported. As shown in Table 2, base rate means for achievement ranged from 0.91 for novels to 1.82 for the *New York Times*, base rate means for reward ranged from 1.04 for novels to 1.86 for Twitter, and base rate means for work ranged from 1.20 for novels to 4.49 for the *New York Times*. These findings support an implication of Study 1, which is that people may commonly focus on achievement, reward, and work when they describe goals, regardless of the regulatory focus or other self-regulatory orientation of the goal they describe. Additionally, it is possible that this finding reflects the fact that the participants in these studies were MTurk workers, who might tend to focus on achievement, reward, and work when doing studies on Mechanical Turk.

## General discussion

When people are in a promotion focus, they tend to view their goals as hopes and aspirations, whereas when they are in a prevention focus, they tend to view their goals as duties and obligations (Higgins, 1997, 1998). Although people could view the goal of a pursuit in any domain of activity as a hope or as a duty, the current research suggests that people's attention often goes to somewhat different activities when they describe these promotion-focused vs. prevention-focused goals. Consistent with research on regulatory focus theory (e.g., Higgins, 1997, 1998; Lee et al., 2000; Aaker and Lee, 2001), differences in language use indicate that participants in the current research paid more attention to positive outcomes when describing hopes than when describing duties, and they paid more attention to social relationships when describing duties than when describing hopes.

Specifically, Study 1 randomly assigned participants to write about a personal experience of pursuing a hope vs. a duty. Linguistic analyses showed that descriptions of pursuing hopes focused more on categorizing the objects, experiences, and other outcomes that participants wanted to gain. Descriptions of hopes also showed a stronger focus on achievement, reward, work, and leisure activities, and they were more positive in tone. In contrast, descriptions of pursuing duties were more in the form of narratives about dynamic social situations in which participants distinguished more between what they actually did and either what they could have done, what they may have wanted to do, or what someone else wanted them to. These descriptions were also moderate in tone.

Study 2 assessed the generalizability of the results of Study 1 to a different form of writing: describing a current hope vs. duty. As in Study 1, linguistic analyses showed that participants' descriptions of hopes were more about positive outcomes, and their descriptions of duties were more about social relationships. However, the hopes and duties these participants brought to mind differed less in their linguistic content than those in Study 1. The descriptions in Study 2 were shorter and more focused on money. Both of these aspects of participants' descriptions could have contributed to the smaller number of linguistic differences between hopes and duties in Study 2 (four significant differences) than in Study 1 (23 significant differences).

A notable similarity between the language in descriptions of hopes and duties in both studies is the high frequency of words about achievement, reward, and work compared to the base rates of these words that Pennebaker et al. (2015b) reported. It is possible that people commonly use these linguistic categories when describing any kind of goal pursuit. Future research could test this hypothesis with promotion and prevention focus, and with other self-regulatory states such as locomotion and assessment (Higgins et al., 2003). Additionally, it is possible that MTurk workers tend to focus on work, achievement, and reward when doing studies on Mechanical Turk, which could result in more frequent use of these words in descriptions by MTurk workers than by participants in other contexts. Future research that assesses limits on the generality of the current findings could examine how much use of words about work, achievement, and reward differ by setting.

The current research has implications for research on regulatory focus and regulatory fit. Studies that vary regulatory focus often ask participants to describe their hopes vs. duties (e.g., Cesario et al., 2004; Vaughn et al., 2006b, 2009, 2010b). If people tend to view certain activities as more relevant to hopes and others as more relevant to duties, then limiting recall of hopes and duties to a specific domain of activity (e.g., leisure or family) could also limit how effectively the recall task varies regulatory focus. This concern is also relevant to studies of regulatory fit, which often ask people to recall hopes vs. duties (e.g., Freitas and Higgins, 2002; Hong and Lee, 2008; for reviews, see Cesario et al., 2008; Vaughn et al., 2010a). An important avenue for future study is to see whether asking people to write about their hopes vs. duties in a specific domain varies regulatory focus as effectively as letting them choose the domains they describe. Additionally, researchers who want to study regulatory focus and regulatory fit within specific domains of goal pursuit may do better to use other ways to vary regulatory focus, such as framing performance tasks in these domains as opportunities to gain vs. maintain positive outcomes (for reviews, see Higgins, 1998; Molden et al., 2007).

### Limits on generality of findings across settings and samples

I expect that in direct replications, the current results will reproduce as long as normative definitions of hopes and duties have not changed. However, although studies with Mturk participants tend to show the same findings as studies with laboratory participants (e.g., Paolacci et al., 2010; Klein et al., 2014), participants in different kinds of settings may differ in their typical hopes and aspirations. For example, college students who write about experiences of pursuing their hopes vs. duties while sitting in a classroom could write much more about academic goals and less about social relationships than participants in the current studies did. Additionally, participants' use of words about achievement, reward, and work could differ depending on whether they are doing the study for pay, for extra credit in their psychology course, or for no external reward.

Another potential limitation to generalizability is that participants resided in the U.S. and Canada. Cultures can differ in assumptions about the personal value of duties and obligations, with people drawing less of a distinction between hopes and duties in collectivist cultures than in individualist cultures (Miller et al., 2011; Cheung et al., 2016). In collectivist cultures, descriptions of duties could be relatively positive and more similar to descriptions of hopes, whereas descriptions of hopes could be more similar to descriptions of duties and focus more on social relationships.

## Conclusion

The current linguistic inquiry extends work on regulatory focus by showing that people do not bring to mind exactly the same domains of activity or personal experiences when describing their hopes and duties. Consistent with earlier work on regulatory focus theory (e.g., Higgins, 1997, 1998; Lee et al., 2000; Aaker and Lee, 2001) people's language use indicates that they focus more on positive experiences when they think about hopes, and they focus more on social relationships when they think about duties. These findings suggest that if researchers are interested in varying regulatory focus with recall of hopes vs. duties, it may help to let participants have a wide range of activities and experiences they can choose to bring to mind.

## Author contributions

The author confirms being the sole contributor of this work and approved it for publication.

### Conflict of interest statement

The author declares that the research was conducted in the absence of any commercial or financial relationships that could be construed as a potential conflict of interest.

## Footnotes

^1^The omitted categories in Study 1 were (1) second-person pronouns, (2) first-person plural pronouns, (3) third-person plural pronouns, (4) anxiety, (5) anger, (6) sadness, (7) hearing, (8) body, (9) sexual, (10) ingestion, (11) risk, (12) religion, (13) death, (14) informal language, (15) swear words, (16) netspeak, (17) assent, (18) nonfluencies, and (19) filler words.

^2^Gender x Regulatory Focus ANOVAs on each category of words in Table 1 revealed no main or interactive effects involving gender that approached *p* < 0.001.

^3^When assessing base rates of word usage, Pennebaker et al. (2015b) only used texts with 25 words or more. Thus, I also analyzed the data from participants whose written descriptions contained 25 words or more (179 in prevention, 152 in promotion; 55.07% of the sample in the main analyses). The average word count in the subsample of participants who wrote 25 words or more was 52.05 (*SD* = 29.51), and it did not differ significantly between conditions, *t*(329) = 0.22, *p* = 0.830. To limit the potential for false-positive results, I kept the same conservative limit for reporting all other results. Specifically, I set the cut-off for significance at *p* < 0.001, two-tailed, with the additional requirement of *d* > 0.35. To summarize the results from this subsample, these analyses showed almost the same significant results as in the main analyses, with four differences. The first was that the prevention condition was no longer significantly higher in words per sentence (*d* = −0.31). The second difference was that the prevention condition was higher in use of words about perceptual feelings such as *feels* and *touch* (*d* = −0.41), which suggests that participants who described duties wrote more about “feeling their way” through complex and not entirely comfortable social situations. The third difference was that the hopes condition was higher in overall use of words about affective processes such as *happy* and *cried* (*d* = 0.42). The affective-processes category of words contains the positive emotion category (*r* = 0.77 with affective processes) and the negative emotion category (*r* = −0.54 with affective processes). Participants in this subsample felt more positive when describing experiences of pursuing hopes, which contributed to the difference in overall use of affective-process words within this subsample. The fourth difference was that participants who described hopes used significantly more words containing six letters or more (*d* = 0.42). Use of big words is part of the analytical/categorical summary variable (Pennebaker, 2011; Pennebaker et al., 2014), and in this subsample it related most strongly to use of words not in the LIWC 2015 dictionary (*r* = −0.34), low use of function words (*r* = −0.35), and more use of words about work (*r* = 0.35).

^4^The second page of Study 2 contained a measure of participants' motivations for pursuing the goal, in order to see whether participants experienced hopes as more self-determined than duties – that is, as more freely chosen (e.g., Deci and Ryan, 2000). This page automatically piped in what the participant wrote on the first page and asked about four kinds of reasons for striving toward the goal (1 = not at all because of this reason, 5 = completely because of this reason): external (“Because somebody else wants you to or because the situation seems to compel it”), introjected (“Because you would feel ashamed, guilty, or anxious if you didn't have this goal”), identified (“Because you really identify with this goal”), and intrinsic (“Because of the enjoyment or stimulation that this goal will provide you”; Sheldon et al., 2004). Consistent with other work (e.g., Sheldon et al., 2004; Milyavskaya et al., 2014), I created a self-determination index, which was the mean of the four items after reverse scoring the items about external and introjected reasons (Cronbach's alpha in the current study = 0.67). Results showed a significant difference between hopes and duties conditions, *t*(335.41) = 8.45, *p* < 0.001, mean difference = 0.81, 95% C.I. [0.62, 1.00], *d* = 0.90. As expected, participants who described a hope or aspiration reported more self-determined motivation to pursue their goal (*M* = 4.05, *SD* = 0.79) than participants who described a duty or obligation (*M* = 3.24, *SD* = 0.99).

^5^The omitted categories in Study 2 were (1) second-person pronouns, (2) first-person plural pronouns, (3) third-person singular pronouns, (4) third-person plural pronouns, (5) negations, (6) interrogatives, (7) anxiety, (8) anger, (9) friend, (10) female, (11) male, (12) seeing, (13) hearing, (14) body, (15) sexual, (16) religion, (17) death, (18) informal language, (19) swear words, (20) netspeak, (21) assent, (22) nonfluencies, and (23) filler words.

^6^Because the lower bound on Cohen's *d* in Study 1 was 0.34, I also examined which linguistic categories showed differences with Cohen's *d*s of 0.34 to 0.40 in Study 2. There was only one such category: total pronouns, *t*(350) = −3.50, *p* = 0.001, mean difference = −3.04, 95% C.I. [−4.75, −1.33], *d* −0.37. As in Study 1, participants in the hopes condition (*M* = 12.13, *SD* = 7.73) used fewer pronouns than participants in the duties condition (*M* = 15.17, *SD* = 8.56). Total use of pronouns correlated most strongly with the analytical/categorical versus dynamic variable (*r* = −0.69), and with function words (*r* = 0.60), including personal pronouns (*r* = 0.91), first-person pronouns (*r* = 0.87), and indefinite pronouns such as *it, it's*, and *those* (*r* = 0.57).

^7^Gender x Regulatory Focus ANOVAs on each category of words in Table 3 and the category of words in Footnote 6 revealed no main or interactive effects involving gender that approached *p* < 0.001.

^8^When assessing base rates of word usage, Pennebaker et al. (2015b) only used texts with 25 words or more. Thus, I also analyzed the data from participants whose written descriptions contained 25 words or more (49 in hopes, 55 in duties; 29.55% of the sample in the main analyses). The average word count in the subsample of participants who wrote 25 words or more was 44.20 (*SD* = 16.04), and it did not differ significantly between conditions, *t*(102) = 0.59, *p* = 0.56. To limit the potential for false-positive results, I kept the same conservative limit for reporting all other results. Specifically, I set the cut-off for significance at *p* < 0.001, two-tailed, with the additional requirement of *d* > 0.40. The only analysis that reached significance was on family; participants who described duties mentioned family more frequently than participants who described hopes (*d* = −0.77).
